# Long-Acting Anti-HIV Drugs Targeting HIV-1 Reverse Transcriptase and Integrase

**DOI:** 10.3390/ph12020062

**Published:** 2019-04-20

**Authors:** Kamal Singh, Stefan G. Sarafianos, Anders Sönnerborg

**Affiliations:** 1 Department of Molecular Microbiology and Immunology, University of Missouri, Columbia, MO 65211, USA; 2 Bond Life Sciences Center, University of Missouri, Columbia, MO 65211, USA; 3 Division of Clinical Microbiology, Department of Laboratory Medicine, Karolinska Institute, Huddinge 14186, Stockholm, Sweden; Anders.Sonnerborg@ki.se; 4 Laboratory of Biochemical Pharmacology, Department of Pediatrics, Emory University School of Medicine, Atlanta, GA 30322, USA; stefanos.sarafianos@emory.edu; 5 Division of Infectious Diseases, Department of Medicine Huddinge, Karolinska Institute, Huddinge 14186, Stockholm, Sweden

**Keywords:** HIV-1, long-acting formulation, reverse transcriptase, integrase, antivirals

## Abstract

One of the major factors contributing to HIV-1 drug resistance is suboptimal adherence to combination antiretroviral therapy (cART). Currently, recommended cART for HIV-1 treatment is a three-drug combination, whereas the pre-exposure prophylaxis (PrEP) regimens consist of one or two antivirals. Treatment regimens require adherence to a once or twice (in a subset of patients) daily dose. Long-acting formulations such as injections administered monthly could improve adherence and convenience, and thereby have potential to enhance the chances of expected outcomes, although long-lasting drug concentrations can also contribute to clinical issues like adverse events and development of drug resistance. Globally, two long-acting antivirals have been approved, and fifteen are in clinical trials. More than half of investigational long-acting antivirals target HIV-1 reverse transcriptase (HIV-1 RT) and/or integrase (HIV-1 IN). Here, we discuss the status and potential of long-acting inhibitors, including rilpivirine (RPV), dapivirine (DPV), and 4-ethynyl-2-fluoro-2-deoxyadenosine (EFdA; also known as MK-8591), which target RT, and cabotegravir (CAB), which targets IN. The outcomes of various clinical trials appear quite satisfactory, and the future of long-acting HIV-1 regimens appears bright.

## 1. Introduction

Revolutionary advances in combination antiretroviral therapy (cART) have rendered HIV from a fatal to chronic disease. If managed efficiently cART significantly improves the life expectancy of a patient [1]. The cART restores (or maintains) CD4 cell counts and suppresses viral load. Currently recommended first-line treatments for HIV infection include a three-drug coformulation in a once-daily fixed-dose single-pill regimen. This formulation virologically suppresses HIV in more than 80% of patients [2,3,4]. Some components have also been recommended for pre-exposure prophylaxis (PrEP) [5]. The approved PrEP regimens are tenofovir disoproxil fumarate (TDF) monotherapy or the combination of TDF and emtricitabine (2’,3’-dideoxy-5-fluoro-3’-thiacytidine or FTC) [5]. The results of clinical trials have shown a reduction in the risk of HIV acquisition by more than 85% in uninfected individuals on PrEP medication [6,7]. In spite of such success, desired therapeutic outcomes of cART are hampered by compromised adherence in both resource-rich and low- and middle-income countries. Long-acting (LA) treatment strategies, especially parenteral, have been successful in facilitating the adherence and minimizing the lapse in medication in other fields of medicine [8,9,10]. Hence, parallel strategies to improve adherence to anti-HIV regimens have been sought. 

To date, one LA antiviral (ibalizumab, a humanized IgG4 antibody) has been approved by the United States Food and Drug Administration. Ibalizumab inhibits HIV infection at post attachment steps by binding to domain 2 of the CD4 receptor and blocking binding of HIV to the cell. Ibalizumab, together with optimized background therapy, is recommended for treatment-experienced patients with multidrug-resistant viruses who are failing current therapy [2]. Another LA drug, which is an entry inhibitor interfering with the binding to gp41 and thereby the fusion step (albuvirtide, a 32-amino acid long analog of gp41) is approved exclusively in China. An additional fifteen antivirals are currently being evaluated in preclinical or advanced clinical trials. Of these, six target HIV-1 reverse transcriptase (HIV-1 RT), two inhibit HIV-1 integrase (HIV-1 IN), three are entry inhibitors, two block capsid (CA) assembly/disassembly processes, and the remaining two inhibit HIV protease (PR). Here, we discuss only RT and IN inhibitors including: EFdA (4-ethynyl-2-fluoro-2-deoxyadenosine) also known as MK-8591, a nucleoside RT inhibitor that inhibits HIV by multiple mechanisms; rilpivirine (RPV), a second-generation non-nucleoside RT inhibitor (NNRTI); and cabotegravir (CAB), a second-generation integrase strand transfer inhibitor (INSTI). 

## 2. LA Antivirals Targeting HIV-1 RT

Currently, three RTIs are in clinical trials and another three are in preclinical development (Table 1). HIV-1 RT is a multifunctional enzyme, composed of two subunits (p66 and p51) that has RNA- and DNA-dependent DNA polymerase activities, as well as an RNase H activity [11]. HIV-1 RT has been the most sought target for anti-HIV drug development. Thus, approximately half of the approved antivirals target RT. There are two classes of HIV-1 RT inhibitors: nucleos(t)ide RT inhibitors (NRTIs) and non-nucleoside RT inhibitors (NNRTIs). The NRTIs are competitive inhibitors that bind at the dNTP (deoxynucleotide triphosphate) binding site [12]. All NRTIs currently approved for the treatment of HIV infection lack a 3’OH and, thus, act as chain terminators [11,12,13]. The NNRTIs are allosteric RT inhibitors, which bind ~10 Å away from the polymerase active site in a pocket known as the NNRTI binding pocket (NNIBP) [14]. The NNRTIs reposition nucleic acid binding [15] and restrict conformational changes required for the catalysis of DNA synthesis by HIV-1 RT [12]. 

### 2.1. Long-Acting NRTIs

#### 2.1.1. EFdA (Islatravir)

**Figure 1:**
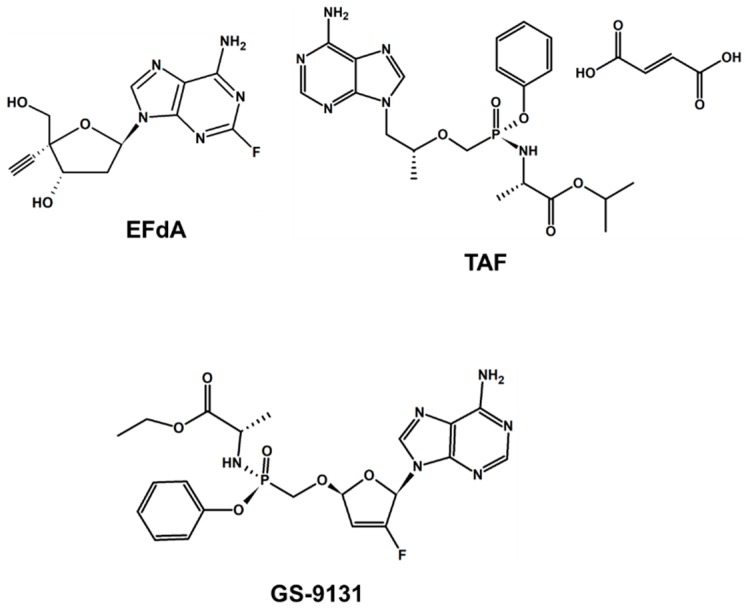
Nucleos(t)ide reverse transcriptase inhibitors in clinical trials or in preclinical development as long-acting (LA) antivirals.

EFdA is an extremely potent NRTI [16]. The EFdA-triphosphate binding affinity of HIV-1 RT can be greater than the natural dNTP substrate [17,18]. In contrast to all approved NRTIs, EFdA retains a 3’-OH group (Figure 1). EFdA also contains a 4’-ethynyl and a 2-fluoro group (Figure 1). Our early studies suggested that incorporation of EFdA-monophosphate in the elongating DNA chain blocks the translocation of RT along the template strand [19,20]. Therefore, we termed EFdA as a translocation-deficient RT inhibitor (TDRTI) [17]. Early molecular modeling [17] and more recent crystallographic studies [20] suggested that the 4’-ethynyl moiety of EFdA binds in a hydrophobic pocket contributing to the strong RT affinity for EFdA. Biochemical studies showed that EFdA inhibits RT through multiple mechanisms: it can block DNA synthesis either as a delayed or as an obligate chain terminator, and EFdA-MP-terminated primers can be protected from excision; also, EFdA-MP is often misincorporated by RT, leading to mismatched primers that are also hard to extend and protected from excision [18,20]. EFdA exhibits picomolar range antiviral activity in activated peripheral blood mononuclear cells (PBMCs) and MT4 cells and can be used in combinations with clinically-used antiretroviral drugs [21]. Importantly, tenofovir-resistant K65R HIV is hypersusceptible to EFdA ([19] and reviewed in [22]). 

EFdA has a high genetic barrier to resistance in culture, which has been attributed to the strong and generally conserved interactions between EFdA and the HIV-1 RT polymerase active site [23,24]. In vitro drug susceptibility assays showed that EFdA had significantly greater efficacy compared to other NRTIs [23]. Moreover, EFdA inhibits both WT and RTI-resistant viruses in a subtype-independent manner [25]. 

With respect to the potential of EFdA/MK-8591 as an LA antiviral, a single injection in rats of an extended release parenteral formulation of MK-8591 (in bioerodible poly(lactic acid) (PLA) and poly(caprolactone) (PCL) and nonerodible ethylene co-vinyl acetate (EVA)) was shown to result in levels of the inhibitor adequate for efficient antiviral effect for more than 180 days [26,27]. EFdA is suitable for formulation in polymer implants [28] and vaginal delivery films [29,30]. Studies have shown a strong potential of EFdA also as a microbicide [30,31]. These studies, combined with studies on macaque animal models [22,32,33], together with the results of phase Ia and Ib human clinical trials [34,35], suggest that EFdA has strong potential as an LA and PrEP regimen or it can be combined with other antiretrovirals for treatment or maintenance therapy.

#### 2.1.2. Tenofovir Alafenamide Fumarate

Tenofovir alafenamide fumarate (TAF) (Figure 1) is chemically related to tenofovir disoproxil fumarate (TDF), which has been part of first-line therapy after its approval in 2001. TDF as monotherapy or co-formulated with emtricitabine (Truvada®) has been approved for PrEP. Reports have shown that a one-tenth dose of TAF has better potency than its predecessor (TDF) [36]. Furthermore, although TAF is more potent than TFV (tenofovir) in vitro, the antiviral susceptibilities to TAF and TFV are highly correlated, indicating that the two compounds have virtually the same resistance profile when assessed as fold change from the wild-type [37]. However, the increased cell loading of TFV with TAF versus TDF observed in vivo suggests that TAF may retain activity against TDF-resistant mutant viruses [37]. Double-blind phase III clinical trial data suggested that TAF-containing regimens have a favorable long-term renal and bone safety profile [38]. A subdermal polyvinyl alcohol implant linearly delivered TFV in plasma at measurable concentrations for more than six weeks [39]. A thin-film polymer devise (TFPD) containing TFV LA demonstrated linear release of 60 days depending on the rate of release [40], and TAF + FTC-loaded nanoparticles showed protection from HIV-1 (Day 4: 80%, Days 7 and 14: 60%, respectively) in humanized BLT (bone marrow-liver-thymus) mice [41]. A release rate of TAF hemifumarate (TAF_2_) ranging between 0.35 and 0.40 mg/day from multipurpose intravaginal rings (IVRs) has also been reported. These rings also contained acyclovir (ACV) and etonogestrel (ENG) in combination with ethinyl estradiol (EE) [42]. In PBMCs, a high concentration of TFV-diphosphate (median, 512 fmol/10^6^ cells) was present up to 35 days. In animal studies, the concentration of TFV-diphosphate was at least 10-fold greater than that associated with effective PrEP in humans taking standard daily doses of oral TDF [39]. 

#### 2.1.3. GS-9131 (Rovafovir Etalafenamide)

GS-9131 (Figure 1) is a phosphonoamidate prodrug with 2’-fluoro modification [43,44] that has demonstrated antiviral activity against viruses containing K65R, L74V, and M184V resistance mutations [42] with a very robust barrier to resistance and a unique resistance profile [43,45]. In early preclinical studies, nucleotide prodrug GS-9131 has shown a favorable toxicity, resistance, and pharmacokinetic profile [46]. An injectable formulation of GS-9131 is currently in preclinical development. The potential of GS-9131 as an LA drug awaits the availability of more clinical results. 

### 2.2. Long-Acting NNRTIs

#### 2.2.1. Dapivirine

**Figure 2:**
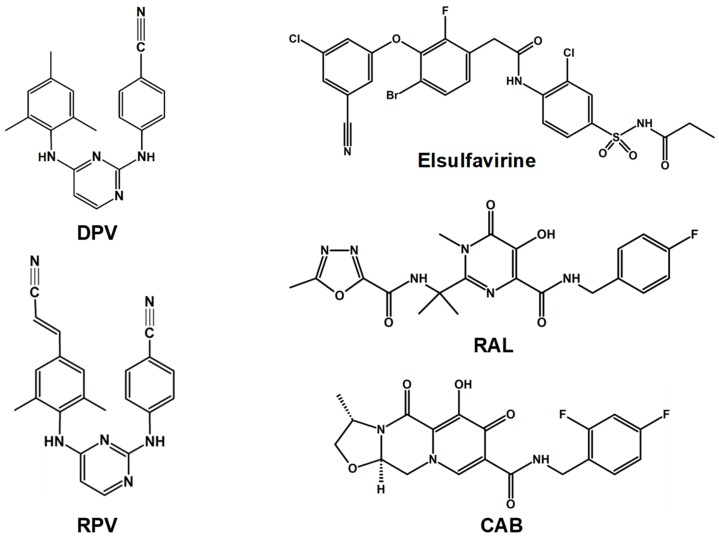
Non-nucleoside reverse transcriptase inhibitors (NNRTIs) and integrase strand transfer inhibitors (INSTIs) in clinical trials and preclinical development as LA antivirals.

Dapivirine (DPV), a diaryl pyrimidine derivative, is a second-generation NNRTI (Figure 2). It is closely related to approved NNRTIs etravirine (ETR) and RPV [47]. DPV is currently under development as a topical microbicide to prevent sexual transmission of HIV in the form of a vaginal ring. The safety and pharmacokinetic profile results of the first clinical trial in a cohort of 16 women who used a DPV-containing vaginal ring for 28 days supported its use as a sustained-release topical microbicide for HIV-1 prevention in women [48]. The vaginal ring contained 25 mg DPV in a platinum catalyzed silicone elastomer matrix [48]. The concentration of DPV in the vaginal fluid on Day 28 exceeded more than 3900-fold IC_99_ in a tissue explant infection model [48]. Another clinical trial included 25 mg DPV or 100 mg maraviroc (MVC) alone or in combination. The inhibition of HIV-1 infection was assessed via ex vivo challenge of cervical tissue samples. The results showed that only DPV had concentration-dependent inhibition of HIV in cervical tissues [49]. The results of the phase III clinical trial ASPIRE (Antiretroviral Strategy to Promote Improvement and Reduce Drug Exposure) showed that the incidence of HIV-1 infection among women who used a silicone elastomer vaginal matrix ring containing 25 mg DPV was decreased by 27% [50]. A follow-up phase III trial (MTN-020/ASPIRE) [51] showed that 19% (6/32) women who acquired HIV-1 during the previous trial experienced virological failure. Results of a number of clinical trials addressing the impact of DPV ring on adherence [52], social concerns [53,54], pharmacokinetics in lactating women [55], pregnancy [56], and ring size [57] have been reported. Several other trials are in advanced stages. The outcomes of reported clinical trials are not necessarily encouraging. Nonetheless, the results of continuing trials could possibly pave the way for the use of the DPV ring as a microbicide.

#### 2.2.2. Rilpivirine

Rilpivirine (RPV) (Figure 2) alone or in combination with cabotegravir (CAB) is one of the most studied antivirals as potential LA agents. RPV is an NNRTI that has been approved in first-line HIV therapy for patients with a viral load of less than 100,000 copies/mL. Phase III clinical trials showed non-inferiority of RPV-containing combination with respect to efavirenz (EFV)-containing therapy [58,59,60]. There are three co-formulations of RPV (TDF/FTC/RPV, TAF/FTC/RPV, and dolutegravir/RPV) available for oral use. 

Encouraging pharmacokinetic properties of RPV LA formulation (in surfactant poloxamer 338) were reported in preclinical and phase I clinical trials [60,61,62,63,64]. These studies prompted phase II clinical trials HPTN (HIV prevention and trial network) 076 [65] and LATTE2 (Long-Acting antireTroviral Treatment Enabling 2) [66]. HPTN 076 was a double-blind, 2:1 randomized trial that was intended to evaluate the safety of 1200 mg of RPV LA compared to placebo [65] in 136 sexually-active and low-risk HIV-uninfected women from Cape Town, South Africa, Harare, Zimbabwe, Newark, New Jersey, and the Bronx, New York. RPV LA was administered in two gluteal, intramuscular injections every eight weeks for a 48-week period, followed by 28-day self-administered 25 mg of daily oral RPV. After withdrawals and discontinuations, 64 women received RPV LA, and 34 received placebo [65]. The results of HPTN 076 showed that RPV LA, 1200 mg intramuscular (IM) every eight weeks was tolerable, safe, and acceptable. RPV plasma concentration at Week 76 was above the protein-adjusted 90% inhibitory concentration (PA-IC_90_) of RPV LA (12.5 ng/mL) in more than 92% of participants [65]. The primary goal of the HPTN 076 clinical trial was the assessment of RPV LA as PrEP.

In the phase IIb LATTE trial, a combination of 25 mg of RPV and selected doses of CAB were used once-daily oral as maintenance therapy for 72 weeks, after an induction period of 24 weeks by CAB with two NRTIs or efavirenz (EFV) plus two NRTIs [67]. The results showed that (i) CAB with 2 NRTIs had potent antiviral activity in the induction phase, and (ii) CAB plus RPV maintenance therapy provided antiviral activity similar to EFV plus two NRTIs until the end of Week 96 [67]. The results of this study demonstrated the potential of RPV LA, which prompted the LATTE2 clinical trial [66]. The LATTE2 trial contained CAB LA and RPV. Since CAB inhibits HIV-1 integrase, a discussion of CAB LA and its potential as an LA agent is discussed in the following section. 

#### 2.2.3. Elsulfavirine

Elsulfavirine (VM-1500) is an NNRTI and prodrug of active compound VM-1500A [68] (Figure 2). It received global approval in Russia for HIV-1 treatment in June 2017. Elsulfavirine has in vitro antiviral efficacy in the nM range (EC_50_ = 1.2 nM), displays antiviral activity against a broad range of NNRTI resistance viruses, and does not have cross-resistance to other NNRTIs [69,70]. Elsulfavirine has shown safety and efficacy comparable to efavirenz (EFV) when combined with TDF and FTC [68]. In a preclinical study, 10 mg/kg injectable elsulfavirine or VM-1500A in beagle dogs showed that VM1500A plasma levels were above 50 ng/mL for at least four weeks, providing a proof-of-concept for its development as an LA antiviral [68]. 

## 3. LA Antivirals Targeting HIV-1 IN

Four INSTIs (raltegravir (RAL), elvitegravir (EVG), dolutegravir (DTG), and bictegravir (BIC)) have been approved for HIV treatment. One of these, RAL and another INSTI cabotegravir (CAB) (yet to be approved) (Figure 2), are at different stages of clinical trials for assessment of their feasibility as LA drugs. As the name implies, integrase strand transfer inhibitors (INSTIs) inhibit HIV-1 IN, a 32-kDa protein that integrates genomic DNA into the host genome. HIV-1 IN functions as an oligomer, which most likely forms upon DNA binding [71,72]. HIV-1 IN has two activities: a 3’-end processing (3’EP) and a strand-transfer (ST) activity. Both activities are conducted by the same active site. Hence, INSTIs, in spite of their nomenclature, also inhibit 3’EP, albeit with low efficacy [73]. 

### 3.1. Cabotegravir

CAB is an investigational second-generation INSTI, which is currently in advanced clinical trials for both treatment and prevention. CAB has a higher resistance barrier than first-generation INSTIs (RAL and EVG), but lower than the other two second-generation INSTIs (DTG and BIC) [74]. A phase IIa clinical trial (HPTN 077) [75] enrolled 200 low-risk healthy individuals in two cohorts. Cohort 1 (*n* = 110) received 800 mg CAB LA intramuscularly (*n* = 82) or placebo (*n* = 28) every 12 weeks after an initial four weeks receiving 30 mg of CAB in mannitol, polysorbate 20, polyethylene glycol 3350, and water once daily. Cohort 2 (*n* = 90) received 600 mg CAB LA intramuscularly (*n* = 69) or placebo (*n* = 20) every eight weeks after receiving 30 mg of CAB for four weeks, once daily. The results of this study revealed that (i) CAB LA was well-tolerated, and (ii) CAB LA 600 mg every eight weeks met pharmacokinetic targets for study participants [75]. 

In the phase IIb LATTE2 clinical trial, 256 HIV-infected treatment-naive patients initially received oral cabotegravir 30 mg plus abacavir (ABC) (600 mg) and lamivudine (3TC) 300 mg once daily for 20 weeks. After the 20-week period, the patients with viral suppression (plasma HIV-1 RNA <50 copies/mL) were randomized in the ratio of 2:2:1 to receive intramuscular CAB LA (400 mg) plus RPV (600 mg) at four-week or eight-week intervals (CAB LA 600 mg plus RPV 900 mg) or the continuation of CAB plus ABC/3TC. The study concluded that injectable combination of CAB LA and RPV every four or eight weeks was as effective as daily CAB/ABC/3TC oral therapy. At Week 96, 84–94% of patients had HIV RNA suppressed to <50 copies/mL. The injectable two-drug combination (CAB LA and RPV) was tolerable and safe [66]. Currently, three Phase III clinical trials are ongoing. These are (i) FLAIR (First Long-Acting Injectable Regimen) (NCT02938520), ATLAS (Antiretroviral Therapy as Long Acting Suppression) (NCT02951052), and ATLAS-2M (NCT03299049). Very recently, the results from ATLAS and FLAIR have been presented. In the ATLAS study, once monthly CAB LA + RPV LA was found noninferior to continued three-drug oral cART at Week 48 and generally well tolerated with infrequent virological failures [76]. In the FLAIR study, monthly injections of CAB+RPV were noninferior to DTG/ABC/3TC at Week 48 and generally well tolerated with few virological failures [77]. Of note is that in a few HIV-1 subtype A1 (HIV-1A1) strains derived from Russian patients failing therapy, primary INSTI mutations had developed. 

### 3.2. Raltegravir

RAL is a first-generation INSTI. It has been recommended by the European AIDS Clinical Society, the United States Department of Health and Human Services, and the International Antiviral Society, USA panel [2,3], as part of PrEP following HIV exposure. A long-acting preparation of RAL (RAL LA) in 5% polyethylene glycol 3350, 0.2% polysorbate 80, and 5% mannitol in water was administered subcutaneously to humanized BLT (bone marrow-liver-thymus) mice and rhesus macaques in a preclinical study [78]. The results showed favorable pharmacokinetic properties in rhesus macaques and potent antiretroviral activity in infected humanized BLT mice together with long-term protection from repeated vaginal HIV challenges in uninfected BLT mice [78]. 

## 4. Challenges of Subtype-Specific Polymorphisms and Pre-Existing Resistance Mutations

Subtype-specific polymorphisms and pre-existing resistance mutations can influence the efficacy of antiretrovirals [14,71,79,80,81]. For example, polymorphism E138A in HIV-1 RT is more common in subtype C (HIV-1C) (6–8%) than HIV-1B (0–2.3%) [82]. Mutation E138A reduces the susceptibility of RPV to varying degrees [82,83,84], leaving the possibility that RPV LA formulation in HIV-1C patients may not yield the desired outcome. 

A detailed phylogenetic analyses showed two distinct genetic clusters in *pol*, which were also maintained in *gag*, *int/vif*, and *env* [85]. These two clusters were linked to either C181 or Y181 in RT, suggesting that C181 group O strains are naturally resistant to NNRTIs.

The resistance profile of CAB is still emerging. However, some reports have documented CAB resistance mutations. In a study conducted with SIVmac251-infected rhesus macaques, mutations I31L, Q91R, E92Q/G/M, T97A/I, G106S, G118R, H156G/R, and V172L and a duplication of five residues at position 232 have been implicated in CAB resistance [86,87]. Except E92Q/G/M and G118R, all mutations are unique to CAB resistance [88]. A recent in vitro resistance selection study showed the emergence of H51N, L74M/I, Q146L, Q148R/K S153Y, S147G, and R263K mutation under CAB pressure [74]. Polymorphisms I31L and L74I/M are present to a varying extent in different HIV-1 subtypes [89]. For example, polymorphism L74I has been reported in more than 93% in HIV-1A1 strains from Russia and countries of the former Soviet Union [90,91,92]. Hence, subtype-specific polymorphisms in HIV-1 IN can affect the outcome of the CAB LA formulation. 

The emergence of NRTI resistance mutations in different subtypes can potentially reduce the long-acting formulation of certain NRTIs. For example, the K65R mutation is associated with both TAF and TDF resistance. Reports have shown that the K65R mutation is selected faster in HIV-1C than in HIV-1B and HIV-1A [79,82]. Globally, HIV-1C is the most prevalent subtype. A high prevalence of K65R has been reported in HIV-1C [93,94,95]. Despite a low prevalence of K65R transmitted resistance mutation [96], a recent comprehensive study suggested a substantial potential for onward transmission to uninfected individuals [97]. Hence, these reports emphasize drug resistance surveillance in untreated individuals for an effective TAF LA formulation. In this context, the EFdA LA formulations may provide better results, as K65R viruses are hypersusceptible to EFdA [19].

## 5. Clinical Challenges with LA Anti-HIV Compounds

Although the preclinical and clinical data presented so far are promising, some clinical issues remain to be addressed. These issues include side effects, drug–drug interactions, and pregnancy, and the pharmacokinetics with long-lasting drug concentrations is not only a prerequisite for the promising clinical benefits, but can also lead to the development of drug resistance. The dosing interval must be selected based on the trough of the compound at the end of the injection interval to ensure that it remains above the concentration required to inhibit HIV-1 efficiently in order to avoid selective pressure for the development of drug resistance. Furthermore, even if the injection interval can be defined so that suboptimal drug concentrations are avoided, there is a potential for emergence of viral resistance as drug concentrations decline during protracted periods of sub-therapeutic exposure after ART discontinuation. Thus, even if LA agents could increase adherence in many patients, an advanced risk for resistance development still persists if patients are non-adherent to the schedule of optimized dosing intervals.

## 6. Conclusions

In conclusion, here, we presented the status of potential LA antivirals that target HIV-1 RT and HIV-1 IN. So far, the results from clinical trials appear encouraging, but more data are required before LA antivirals can be commonplace. Nonetheless, the future of the LA formulations appears bright. Once the LA combinations are approved, improved adherence to and convenience of cART are expected to be achieved.

## Figures and Tables

**Table 1:** Long-acting antivirals targeting HIV-1 RT and HIV-1 IN. NRTI, nucleos(t)ide RT inhibitors; NNRTI, non-nucleoside RT inhibitors; INSTI, integrase strand transfer inhibitors; EFdA, 4-ethynyl-2-fluoro-2-deoxyadenosine; TAF, tenofovir alafenamide fumarate; DPV, dapivirine; RPV, rilpivirine; RAL, raltegravir.

Drug Class	Drug	Formulation	Clinical Trial Stage
NRTI	EFdA	Implant (vaginal film, subcutaneous polyethylene vinyl acetate membrane)	Phase II
	TAF	Implant (multipurpose intravaginal ring, subdermal polyvinyl acid membrane, subcutaneous thin-film polycaprolactone) Injectable (subcutaneous nanosuspension)	Preclinical
	GS-9131	Injectable (intravenous propylene or polyethylene glycol in citric acid)	Preclinical
NNRTI	DPV	Implant (vaginal ring)	Phase III
	RPV	Injectable (subcutaneous/intramuscular nanosuspension) Implant (microarray patch) Topical (nanoformulation)	Phase III
	Elsulfavirine	Injectable (subcutaneous/intramuscular nanosuspension)	Preclinical
INSTI	CAB	Injectable (intramuscular nanosuspension)	Phase III
	RAL	Injectable (subcutaneous nanosuspension)	Preclinical
